# Effects of an Eight-Week Psychomotor Processing Speed Program on Physical and Cognitive Abilities in Community-Dwelling Older Adults

**DOI:** 10.1177/00315125251359748

**Published:** 2025-07-10

**Authors:** Luis Galhardas, Hélio Mamoru Yoshida, Armando Raimundo, José Marmeleira

**Affiliations:** 1 Departamento de Desporto e Saúde, Escola de Saúde e Desenvolvimento Humano, Universidade de Évora, Évora, Portugal; 2 Comprehensive Health Research Centre (CHRC), Évora, Portugal; 3 Departamento de Ciências da Saúde, 74348 Universidade Federal do Triângulo Mineiro– UFTM, Uberaba, Brazil

**Keywords:** psychomotor, elderly, physical performance, cognitive functioning

## Abstract

The aim of this study was to examine the effects of a psychomotor processing speed program on the physical and cognitive functioning of community-dwelling older adults. Twenty-two participants (80.6 ± 1.9 years) were allocated to the exercise group (EG) while twenty-one (mean age: 79.2 ± 1.2 years) were designated to the control group (CG). Participants in the exercise program underwent a psychomotor processing speed intervention twice a week for a duration of 8 weeks. Evaluations were conducted at baseline and post-intervention. Following the 8-week program, statistically significant enhancements (*p* < .05) were observed in all physical fitness parameters (strength, cardiorespiratory endurance, flexibility, and balance), as well as in most cognitive parameters (reaction time, visual attention, executive functioning, and processing speed). Small to large effect sizes were established. The findings suggest that a psychomotor processing speed program can have a comprehensive impact on an individual’s functional abilities and should be promoted for implementation in community-dwelling older adults.

## Introduction

Presently, across a significant portion of the world, the majority of individuals can anticipate living beyond the age of 70 (World Health Organization, 2015). While increasing average life expectancy is crucial, the emphasis should also be on ensuring a prolonged life characterized by quality. Older adults often experience various limitations, including physical aspects such as aerobic capacity, muscular strength, and balance (Chmelo et al., 2015; Germain et al., 2016; Marmeleira et al., 2018), as well as cognitive capacities like memory and information processing (Agmon et al., 2014; Chmelo et al., 2015; Håkansson et al., 2016). Moreover, the aging process is frequently characterized by feeling of loneliness and an increased reliance on others, alongside with declines in overall health and physical and cognitive function (Choi et al., 2017).

Physical function refers to the capacity to carry out essential activities of daily living and instrumental activities of daily living, which are crucial for maintaining independence and significantly influence quality of life (Ahmad et al., 2022; Sheng et al., 2022). Physical activity can induce several benefits for older adults. Research indicates an inverse association between physical activity and all-cause mortality in older men and women (Brown et al., 2012). Additionally, consistent engagement in physical activities and maintenance of an active lifestyle contribute to preserving health and enhancing physical function among older adults (Lee et al., 2016). Participation in physical activities and the preservation of functional ability are pivotal factors for achieving successful aging (Marini et al., 2015; Resnick et al., 2015). Older adults who regularly participate in physical activity often perceive themselves as having undergone successful aging (Resnick et al., 2015).

The age-related changes that arise over the years, lead to an inevitable decline in the performance of activities of daily living, which compromises autonomy and independence (Izquierdo et al., 2016; Rosset et al., 2011). However predictable, the loss of capabilities due to advancing age can be prevented, mitigated, or even treated through appropriate stimulation of physical capacities, including strength, balance, walking, and flexibility, among others (Jiménez-García et al., 2019; Martínez-Amat et al., 2013). Consistent engagement in physical exercise has the potential to augment functional capacity, enhance mobility, and, consequently, foster autonomy (Jiménez-García et al., 2019).

In recent years, several studies have focused on assessing the impact of various intervention programs on the functional capacities of older adults, including those involving combined cognitive and exercise interventions. This approach has demonstrated significant relevance, given its potential to enhance both general physical capacities and cognitive functioning (Marmeleira et al., 2018; Rosado et al., 2021). Typically, interventions that integrate cognitive and physical exercises are implemented either sequentially or simultaneously, often within dual-task paradigms (Law et al., 2014). Cognitive function refers to the brain’s ability to recognize and interpret objective information. It encompasses various mental abilities, including perception, attention, memory, thinking, and language (Liu et al., 2024; Wang et al., 2022).

Integrating cognitive and physical exercises has the potential to activate various mechanisms, including neuroplasticity and cerebral blood flow, which underlie the positive influences of physical activity on cognition and the brain (Cassilhas et al., 2016; Di Liegro et al., 2019). If exercise not only demands physical skills but also involves information processing (perceptual and cognitive components), it is reasonable to consider that training over time could impact functional status (Marmeleira, 2013). Recent findings indicate that physical activity may contribute to preserving reasoning, speed, and vocabulary abilities in the presence of white matter hyperintensity burden (Song et al., 2022). This line of research is aligned with the idea that age-related variations in cognitive function may stem from a generalized slowing of processing speed associated with aging (Birren & Fisher, 1995).

Processing speed (PS) refers to the time required for individuals to process and perform cognitive tasks, particularly elementary cognitive tasks (Takeuchi & Kawashima, 2012). In a literature review, it was suggested that a specific form of computer-based training, focused on the speed of information processing (processing speed training), holds promise for enhancing cognition and improving everyday activities in older adults (Ball et al., 2007). Subsequent investigations propose that processing speed training is an intervention with significant potential at both cognitive and psychomotor levels (Nouchi et al., 2016; Rebok et al., 2014; Takeuchi et al., 2011). Recently, a 12-month Randomized Controlled Trial suggested that improved processing speed could be a mechanism through which exercise reduces injurious falls in older adults who have experienced falls previously (Liu-Ambrose et al., 2021).

As mentioned earlier, intervention programs that integrate both physical and cognitive activities could significantly impact an individual’s level of functioning. Such programs have the potential to activate multiple mechanisms that contribute to the positive effects of physical activity on various aspects, including physical fitness, health risk factors, and brain function. Therefore, based on the processing speed theory (Salthouse, 2000; Ticha et al., 2023) and existing knowledge about the efficacy of multimodal programs, the present study aimed to investigate the effects of a psychomotor-based processing speed intervention program on the physical and cognitive functioning of older adults living in the community. Specifically, our hypothesis posits that various cognitive abilities such as reaction time, information processing speed, visuospatial ability, selective and sustained attention, along with physical fitness components like body strength, aerobic endurance, flexibility, and balance, would experience positive benefits through participation in the psychomotor intervention.

## Methods

### Trial Design

A non-randomized controlled trial, comprising an 8-week intervention was conducted. The intervention program was delivered in groups, with a maximum of 8 participants in each group, resulting in a total of 3 groups. All groups engaged in similar exercises.

### Participants

The participants, both in the experimental and control groups, were community-dwelling older adults recruited from social centers in the Évora region of Portugal. The G*Power software determined that a minimum sample size of 21 participants per group, totaling 42 participants, was necessary, with a significance level (α) of 0.05 and a power of 0.80. The positive benefits for physical fitness were the primary outcome that the study power was designed to investigate. Inclusion criteria comprised: (1) Community-dwelling older adults of both sexes aged ≥65; (2) Absence of physical disabilities or other chronic health conditions that could hinder participation in the intervention program; (3) No engagement in any exercise program for at least six months; and (4) Normal cognitive status as assessed by the Portuguese version of the Mini-Mental State Examination (Folstein et al., 1983; Guerreiro et al., 1994; Morgado et al., 2009).

Fifty-six older adults were invited to participate in the study, with 13 declining to take part. Subsequently, forty-three older adults who met the inclusion criteria were enrolled by convenience into the exercise group (*n* = 22, EG) and the control group (*n* = 21, CG). Randomization was not feasible due to logistical constraints associated with session scheduling and the availability of intervention spaces. For example, the considerable geographical distance between participating institutions necessitated the assignment of some to the exercise group and others to the control group.

Table 1 illustrates the general characteristics of the participants. The sample comprised participants of both sexes aged 65–92 years. Overall, the participants had a low educational level, with 37.2% never attending school. According to the World Health Organization criteria for body mass index, approximately 35% of the participants were classified as overweight (≥25 kg.m^2^), and 21% were categorized as obese (≥30 kg.m^2^). Additionally, the Katz Index (Katz et al., 1963; Sequeira, 2007) was applied, revealing that the majority of participants were independent in performing basic activities of daily living. No statistically significant differences (*p* ≤ .05) were observed between the experimental group (EG) and control group (CG). After completing the initial study, participants in the control group were offered the opportunity to take part in an exercise program similar to the one conducted during the study.

**Table 1.** Overview of Participant Characteristics.

	EG (*n* = 22) Mean (SD)	CG (*n* = 21) Mean (SD)	*p*-value*
Age (years)	80.59	79.24	0.31
Sex, female (%)	14 (63.6)	12 (57.1)	0.67
Height (cm)	161.23	165.19	0.13
Weight (kg)	68.05	71.99	0.36
BMI (kg/m^2^)	26.08	26.43	0.81
Education (years)	4.05	3.24	0.73
MMSE (points)	25.50	26.52	0.55
Katz scale (points)	5.77	6.00	0.16

*Note.* MMSE, Mini-Mental State Examination; BMI, body mass index; SD, standard deviation; significant differences within groups, *p* ≤ .05; EG, exercise group (*n* = 22); CG, control group (*n* = 21). *Mann-Whitney U test.

All participants were informed about the objectives of the study and signed an informed consent before participation.

### Procedures

Prior to data collection, the research team, under the guidance of a senior researcher experienced in this field, familiarized themselves with all assessment methods. All the participants were assessed individually, in a peaceful area, at the baseline and after eight weeks of intervention. The experimental group underwent a total of 16 intervention sessions (twice a week); additional sessions were occasionally necessary to compensate for some participants’ absences. Additionally, approximately one week before the initial assessment, all participants underwent a training trial session to acquaint themselves with the assessment methods.

The assessment measures encompassed cognitive and physical functions. Sociodemographic characteristics was also collected through a questionnaire. Consistency was maintained by having the same evaluator administer each assessment method, providing task demonstrations before each trial.

The height of participants (cm) was measured without shoes using a wall-mounted tape, and their weight (kg) was assessed with a mechanical scale (Seca 750, Hamburg, Germany). The body mass index (BMI) was calculated by dividing the weight in kilograms by the square of the height in meters. Additionally, the Katz Index, a tool for evaluating an individual’s level of independence in daily living activities, was applied (Katz et al., 1963).

## Outcome Measures

### Physical Fitness

The Senior Fitness Test Battery (SFT) (Rikli & Jones, 2013) was utilized to assess lower- and upper-body strength, range of motion, and aerobic endurance. The following tests were administered: 30 s chair stand, arm curl, chair sit-and-reach, back scratch, and 6 minutes’ walk. Agility was measured using the 3 m timed up and go test (TUG) under both single- and dual-task conditions (TUG-DT). In the dual-task conditions, participants were instructed to count down from 50 as quickly and accurately as possible while performing the motor task (Marmeleira et al., 2018). This assessment method has demonstrated reliability and validity in estimating fall risk among healthy elderly individuals (Hofheinz & Schusterschitz, 2010).

Furthermore, we employed the Short Physical Performance Battery (SPPB), which integrates the outcomes of gait speed, chair stand, and balance tests (Guralnik et al., 1994). The balance assessments comprised three progressively challenging tasks involving different feet positions (feet together stand, semi-tandem, and full tandem stand). To advance to the next position, the individual needed to maintain stability for 10 seconds in each stance. Gait speed was evaluated through the 4-m walk test, recording the time taken to cover the specified distance. The five times sit-to-stand test (chair stand test) began with participants seated. Following confirmation of their ability to complete one sit-to-stand action independently, participants were directed to perform five repetitions as rapidly as possible while ensuring their feet remained flat on the floor. SPPB scores were determined based on performance, with points awarded for each successfully completed task. The maximum overall score was 12 points, distributed evenly among the four components.

The Arm Ruler Positioning Test (ARPT) was employed for the assessment of upper limb proprioception (Galhardas et al., 2020). In the ARPT, participants were tasked with responding to various finger and arm positions. In brief, the kinesiologist gradually moved the participant’s finger to the specified target position, maintaining it for 5 seconds. Subsequently, the kinesiologist passively returned the participant’s finger to the starting position. The participant then actively attempted to reproduce the target position, signaling the kinesiologist when they believed they had successfully replicated the previous position (Galhardas et al., 2020). The outcome measurement was the error score, calculated as the mean absolute difference in centimeters between the target position and the actual position of the index finger over eight repetition attempts.

### Cognitive Functioning

We conducted evaluations encompassing processing speed, reaction time, attention, memory, and inhibitory function. For assessing processing speed, we opted for the Trail Making Test – Part A (TMT-A). In this test, participants are tasked with sequentially connecting, in ascending order, 25 encircled numbers randomly distributed on a sheet of paper (Reitan, 1958). The score was determined by the time (in seconds) taken to accurately complete the task, and the number of errors made was also recorded. TMT-A assesses visual scanning, graphomotor speed, and executive functioning (Llinàs-Reglà et al., 2015).

We evaluated simple reaction time (SRT) in both single and dual-task conditions, along with choice reaction time (CRT), using the Deary–Liewald Reaction Time Task (DLRT). This task employs a computer program and has been proven to offer reliable and valid procedures across a broad age range (Deary et al., 2011; Ferreira et al., 2021). In the SRT component, participants were required to press the space bar in response to a stimulus (a cross appearing inside a square). We also introduced a dual-task assessment, where participants had to verbally report the results of simple mathematical calculations presented by the researcher while simultaneously pressing the space bar in response to stimuli. These calculations were uniform and presented in the same order for all participants. For the CRT, four crosses appeared on the computer screen, and participants were instructed to press the button corresponding to the correct response. Median reaction time (in milliseconds) was recorded for each participant in each task. Additionally, the total number of errors (pressing the wrong key) in the CRT was documented. This methodology aligns with a similar approach employed previously (Marmeleira et al., 2018).

The go/no-go task involves a participant generating a simple motor response to one signal while refraining from responding to another signal (Verbruggen & Logan, 2008). This task serves to measure both reaction time and impulsiveness (inhibition). To implement this assessment, we utilized a computer program, specifically the PsyToolkit (Stoet, 2010, 2017). During the task, the participant was prompted to respond within 2 seconds by clicking the space bar when the “Go” signal was presented in green. Conversely, when the “No-go” signal appeared in red, the participant was instructed to refrain from any action, meaning they should not press the button for the duration of 2 seconds.

The Eriksen flanker task is a conventional method employed to assess the impact of task-irrelevant information on the processing of task-relevant information. In this task, participants are tasked with responding to a visual target element surrounded by task-irrelevant elements (Eriksen & Eriksen, 1974; Ulrich et al., 2021). To elaborate, the target is flanked by non-target stimuli that either correspond to the identical directional response as the target (congruent flankers) or to the opposite response (incongruent flankers). A directional response (left or right) is assigned to a central target stimulus. Typically, response times tend to be longer for incongruent attempts, partly because they require the additional step of focusing attention (Servant & Logan, 2019). This assessment method was also implemented using a computer program (PsyToolkit) (Stoet, 2010, 2017). The score for the tasks was based on mean response times (in seconds) in correct performances.

Attention was assessed using the d2 Test of Attention, a neuropsychological measure specifically designed to evaluate both selective and sustained attention (Brickenkamp, 2007). Participants were instructed to identify and mark the letter ‘d' with two apostrophes. The scores included the number of items processed (indicating the performance on all processed items), the number of items recognized correctly, total errors, the percentage of total errors, and total efficacy (calculated as the number of items processed minus total errors).

The Montreal Cognitive Assessment (MoCA) is known for its heightened sensitivity in identifying cognitive decline (Freitas et al., 2011; Nasreddine et al., 2005). In this research, we focus on a specific facet of the MoCA, namely working memory. Points are allocated based on the accuracy of responses, serving as a metric for evaluating an individual’s cognitive performance.

### Psychomotor Processing Speed Program

Participants enrolled in an 8-week training program, involving 45-min sessions conducted twice a week in small groups (approximately 6 participants), under the supervision of a trained kinesiologist. The innovation in our processing speed training lies in its fusion of traditional cognitive processing speed training with movement. This approach incorporates relatively simple motor, physical, and reaction time activities, strategically designed to exert pressure on physiological, perceptual, and cognitive mechanisms. The training sessions were meticulously planned to ensure the provision of stimuli pertinent to physical fitness, cognition, and, notably, processing speed.

The sessions were structured into three phases: mobilization (5 minutes), main phase (35 minutes), and cool down (5 minutes). The initial mobilization aimed to activate crucial physiological systems and included simple exercises such as range of motion and stretching movements. In the main phase, specific intervals lasting 5–10 minutes (3/4 segments) were allocated to exercises primarily targeting processing speed (examples provided in Table 2). Additionally, to psychomotor speed, these exercises encompassed cardiovascular, strength, balance/agility, and flexibility activities. Participants were consistently assigned dual tasks, underscoring the significance of time or speed in task execution. The final phase of the sessions incorporated stretching, breathing, and relaxation movements to guarantee a thorough cooldown.

**Table 2.** Illustrative Activities in Psychomotor Processing Speed Training.

Activities
1. While walking, participants respond to various auditory or visual cues, requiring quick and specific psychomotor reactions (e.g., closing hands, touching index and thumb fingertips together, joining hands, clapping)
2. While standing, participants are tasked with rapidly sorting straws according to pre-established colors
3. While seated back-to-back, participants, paired by name, were required to swap places as swiftly as possible
4. While the participants maintain a number of balloons (different colours) in the air, they are required to swiftly grasp a balloon of a specific color
5. Participants are instructed to mimic the gestures of the kinesiologist, excluding specific gestures that have been pre-defined
6. Employing predetermined movements, participants promptly responded to numbers spoken aloud by the kinesiologist. For instance, “one” corresponded to a squat, “two” to a step back, and “three” to a stepping exercise
7. Utilizing tangram, participants are required to complete the suggested models swiftly while concurrently engaging in squats or dumbbell exercises (using one hand)
8. As participants move through the space, each one carries a distinct object, and the kinesiologist prompts them to exchange specific objects as rapidly as possible
9. While executing squats, participants concurrently engaged in rapid mental arithmetic, alternating between addition and subtraction with each repetition, aiming for one calculation per squat
10. During the execution of movements using the training band for both upper and lower limbs, participants engaged in category recall exercises. The objective was to swiftly recall items within a category, one by one, without duplicating any names already mentioned by other participants. Example categories included animals, proper names beginning with a specific letter, and others

The sessions were structured to establish a secure and gradual exercise regimen, with the complexity of the assigned tasks escalating throughout the program. The sessions were planned to range from moderate to intense in intensity, and Borg’s subjective effort scale (Borg, 1990) was employed to recalibrate them as needed. The nature of activities was also adapted based on satisfaction results gauged through an analog scale ranging from 1 to 5 (Likert scales).

The sessions were planned and executed by a kinesiologist holding a degree in psychomotor rehabilitation, under the supervision of a senior researcher specialized in exercise and health, and psychology. All sessions were planned to be consistent across all groups.

### Data Analysis

The normality and homogeneity assumptions were assessed using the Shapiro–Wilk test. Since the variables exhibited a normal distribution, parametric statistical analyses were applied. Between-group comparisons were conducted using the independent-sample *t*-test, while within-group comparisons utilized the paired *t*-test. Descriptive analysis, incorporating mean and standard deviation, was employed for all data.

The delta value (Δ%) between each assessment moment (pre-test and post-test) was computed using the formula: Δ% = [(post-test−pre-test)/pre-test] × 100. ANCOVA was conducted to assess the group effects of the multimodal intervention in the post-test, adjusting for the pre-test score. The partial eta squared (η^2^) value in the ANCOVA tests serves as an indicator of effect sizes based on Cohen’s guidelines (Cohen, 2013), with cutoff values of 0.01, 0.06, and 0.14 denoting small, medium, and large effects, respectively. Additionally, we also compute the effect size for paired comparisons using Cohen’s method (Cohen, 2013): (post-test mean - pre-test mean) / pre-test standard deviation. An effect size of <0.3 is considered small, 0.30 to 0.80 is medium, and >0.80 is large.

For all statistical tests, the level of significance was *p* < .05. Data analysis was conducted using the SPSS statistical program (version 20.0, Inc, Chicago, IL, USA).

## Results

There were no significant differences between the groups regarding physical and cognitive functioning at the baseline. The intervention targeting psychomotor processing speed resulted in several positive effects on both cognitive and physical variables. Table 3 illustrates the comparisons between and within the groups, indicating a significant improvement in various physical aspects (*p* < .05). Following the 8-week intervention period, no statistically significant changes were detected in the control group (CG). After the same period, the exercise group exhibited significantly better performances in various components of the *SFT* - chair stand test (Δ%: 23, *p* < .001); arm curl test (Δ%: 17, *p* < .001); back scratch (Δ%: 4, *p* < .007); 6-min walk test (Δ%: 15, *p* < .001); Agility – timed up and go test (Δ%: −11, *p* < .001); Dual-task timed up and go test (Δ%: −10, *p* < .001); *SPPB* – overall points (Δ%: 12, *p* = .002); Repeated chair stand (Δ%: −15, *p* = .002); gait speed (Δ%: 9, *p* < .001). Regarding physical assessment, only flexibility of the lower limbs (SFT - chair sit and reach test) and proprioception (ARPT) did not exhibit statistically significant changes within the exercise group.

**Table 3.** Impact of the Intervention Program on Physical Fitness.

	Baseline (A) (Mean ± SD)	Post-intervention (B) (Mean ± SD)	Adjusted means, M (95%CI)	p^a^	ηp2
Senior fitness test					
Chair stand (rep)					
EG	7.77 ± 3.64	10.14 ± 3.93*	2.36 (2.01; 2.71)	0.01	0.66
CG	9.48 ± 2.75	9.23 ± 2.7	−0.14 (−0.71; 0.24)		
Arm curl (rep)					
EG	9.91 ± 4.16	12.00 ± 3.95*	2.09 (1.24; 2.94)	<0.01	0.28
CG	9.67 ± 3.17	9.95 ± 3.22	−0.29 (−0.19; 0.77)		
Chair sit and reach (cm)					
EG	−17.41 ± 15.18	−16.90 ± 14.10	0.51 (−0.42; 1.44)	0.03	0.10
CG	−19.48 ± 11.62	−20.02 ± 12.04	−0.55 (−1.14; 0.47)		
Back scratch (cm)					
EG	−33.01 ± 13.92	−31.61 ± 13.15*	2.20 (0.43; 2.39)	0.01	0.16
CG	−31.11 ± 14.72	−31.41 ± 14.88	−0.30 (−1.09; 0.49)		
6 min walk (m)					
EG	269.09 ± 105.49	314.77 ± 107.18*	45.68 (33.18; 58,18)	<0.01	0.45
CG	266.43 ± 84.66	265.43 ± 91.18	−1.00 (−12.31; 10.31)		
Agility					
TUG (s)					
EG	15.03 ± 5.46	13.54 ± 4.38*	−1.49 (−2.16; −0.82)	<0.01	0.44
CG	15.37 ± 3.89	15.35 ± 3.86	−0.03 (−0.25; 0.19)		
TUG-DT (s)					
EG	15.23 ± 5.22	13.87 ± 4.63*	−1.36 (−1.77; −0.95)	<0.01	0.61
CG	15.62 ± 3.71	15.67 ± 3.72	0.05 (−0.09; 0.20)		
Short physical performance battery					
SPPB (points)					
EG	6.55 ± 2.56	7.41 ± 2.65*	0.86 (0.43; 1.30)	<0.01	0.25
CG	7.00 ± 2.21	6.95 ± 2.29	−0.05 (−0.27; 0.18)		
Repeated chair stand (s)					
EG	17.59 ± 6.95	15.59 ± 5.11*	−2.00 (−3.40; −0.60)	0.01	0.16
CG	15.25 ± 4.22	15.45 ± 4.60	0.20 (−0.15; 0.56)		
Gait speed (m/s)					
EG	0.65 ± 0.13	0.71 ± 0.14*	0.06 (0.04; 0.09)	<0.01	0.56
CG	0.67 ± 0.15	0.66 ± 0.14	−0.01 (−0.01; 0.17)		
Proprioception					
Arm ruler positioning test					
EG	4.26 ± 1.02	4.10 ± 0.86	−0.16 (−0.39; 0.07)	0.02	0.12
CG	4.33 ± 0.53	4.40 ± 0.55	0.08 (−0.01; 0.17)		

*Note.* SD, standard deviation; EG, exercise group (*n* = 22); CG, control group (*n* = 21); *p < .05 changes within the group by Paired *t*-test; ^a^ *p*-value ANCOVA; η_p_^2^, partial eta-squared; CI, confidence interval; TUG, timed up and go test; SPPB, short physical performance battery.

In general, participants in the exercise group demonstrated improved strength, flexibility, aerobic capacity, agility, balance, and gait speed following the program. ANCOVA analysis revealed significant differences between groups after the intervention program in all analyzed physical components. The computed partial eta-square (η_p_^2^) indicated medium to large effect sizes in the physical performance variables.

Additionally, for paired comparisons, the effect sizes in physical fitness for the exercise group were as follows: 0.65 for ‘Chair Stand’, 0.50 for ‘Arm Curl’, <0.1 for ‘Chair Sit and Reach’, 0.11 for ‘Back Scratch’, 0.43 for ‘6-Minute Walk’, 0.27 for ‘TUG’, 0.26 for ‘TUG-DT’, 0.34 for ‘SPPB’, 0.29 for ‘Repeated Chair Stand’, 0.46 for ‘Gait Speed’, and 0.16 for the ‘Arm Ruler Positioning Test’.

In cognitive performance (Table 4), following the 8-week intervention period, within-group analysis revealed that the control group (CG) maintained consistent cognitive performance without statistically significant changes. Conversely, the experimental group (EG) exhibited improved performance in various tests, particularly in processing speed (TMT-A), reaction time (go no go and DLRT), and attention (d2). Hence, the experimental group (EG) demonstrated significantly improved performances in the Trail Making test -part A – assessment time - (Δ%: −9, *p* = .002); Go no go test – reaction time (Δ%: −11, *p* = .001); errors (Δ%: −50, *p* = .007); D2 test – items processed (Δ%: 13, *p* < .001); items recognized correctly (Δ%: 11, *p* = .001); errors (Δ%: −12, *p* = .007); total efficacy (Δ%: 14, *p* < .001); DLRT - SRT (Δ%: −7, *p* < .001); SRT-DT (Δ%: −7, *p* = .002); mental calculations (Δ%: 7, *p* = .003); and CRT (Δ%: −8, *p* < .001). In the MOCA (memory) and Eriksen Flanker Task (flanker effect), there were no statistically significant alterations in the EG.

**Table 4.** Impact of the Intervention Program on Cognitive Functioning.

	Baseline (A) (Mean ± SD)	Post-intervention (B) (Mean ± SD)	Adjusted means, M (95%CI)	p^a^	ηp2
TMT-A					
TMT-A (s)					
EG	73.29 ± 30.09	67.18 ± 27.02*	−6.12 (−9.35; −2.88)	0.01	0.22
CG	76.01 ± 28.55	76.26 ± 30.27	0.25 (−2.15; 2.65)		
TMT-A errors (*n*)					
EG	2.27 ± 1.96	1.82 ± 1.56	−0.45 (−1.01; 0.11)	0.42	0.01
CG	2.14 ± 1.71	1.95 ± 1.56	−0.19 (−0.53; 0.15)		
MOCA					
Memory					
EG	3.27 ± 0.70	3.45 ± 0.67	0.18 (−0.08; 0.44)	0.43	0.02
CG	3.38 ± 0.59	3.43 ± 0.51	0.05 (−0.05; 0.15)		
Eriksen flanker task					
Flanker effect (ms)					
EG	189.14 ± 83.01	165.32 ± 78.53	−23.82 (−62.27; 14.63)	0.06	0.09
CG	213.95 ± 76.70	214.67 ± 69.47	0.71 (−13.92; 15.35)		
Go no go test					
Reaction time (ms)					
EG	728.38 ± 134.65	656.38 ± 114.99*	−71.99 (−107.86; −36.14)	<0.01	0.30
CG	706.42 ± 89.90	711.50 ± 83.31	5.08 (−12.31; 22.47)		
Errors (*n*)					
EG	1.23 ± 0.81	0.82 ± 0.67*	−0.41 (−0.67; −0.15)	0.10	0.07
CG	1.00 ± 0.71	0.91 ± 0.70	−0.10 (−0.29; 0.10)		
d2 test					
Items processed (*n*)					
EG	116.32 ± 53.48	133.23 ± 59.84*	16.91 (10.33; −23.49)	<0.01	0.30
CG	124.95 ± 44.05	121.71 ± 37.37	−3.24 (−10.63; 4.15)		
Items recognized correctly (*n*)					
EG	45.50 ±22.83	51.00 ± 24.60*	5.50 (1.42; 9.58)	0.01	0.16
CG	49.57 ± 20.25	47.86 ± 17.16	−1.71 (−4.83; 1.40)		
Errors (%)					
EG	20.07 ± 11.03	17.98 ± 9.24*	−2.10 (−3.41; −0.79)	0.03	0.12
CG	20.92 ± 9.61	20.33 ± 9.35	−0.59 (−1.62; 0.43)		
Total efficacy (*n*)					
EG	95.13 ± 50.24	111.03 ± 55.03*	15.90 (10.02; 21.78)	<0.01	0.31
CG	101.40 ± 45.56	98.99 ± 39.12	−2.41 (−8.95; 4.13)		
DLRT					
SRT (ms)					
EG	492.50 ± 122.65	460.96 ± 106.84*	−31.55 (−47.16; −15.93)	<0.01	0.24
CG	492.43 ± 55.31	487.33 ± 53.80	−5.10 (−12.92; 2.73)		
SRT-DT (ms)					
EG	583.05 ± 132.77	543.96 ± 124.96*	−39.09 (−63.80; −14.39)	<0.01	0.25
CG	561.38 ± 68.73	572.14 ± 70.33	10.76 (−0.22; 21.75)		
Mental calculations (*n*)					
EG	17.59 ± 6.33	18.95 ± 6.31*	1.36 (0.60; 2.13)	0.04	0.11
CG	18.76 ± 4.11	19.10 ± 4.38	0.33 (−0.21; 0.88)		
CRT (ms)					
EG	1000.91 ± 249.91	930.00 ± 221.14*	−70.91 (−115.25; −26.57)	<0.01	0.29
CG	1026.91 ± 114.40	1036.10 ± 117.67	9.19 (−4.75; 23.13)		

*Note.* SD, standard deviation; EG, exercise group (*n* = 22); CG, control group (*n* = 21); *p < .05 changes within the group by Paired T Test; ^a^, *p*-value, based on ANVOCA; η_p_^2^ , partial eta-squared; CI, confidence interval; TMT, trail making test; MOCA, Montreal Cognitive Assessment; DLRT, Deary-Liewald Reaction Time task; SRT, simple reaction time; CRT, choice reaction time.

ANCOVA analysis indicated significant differences between groups after the intervention program in several analyzed cognitive components, specifically in processing speed (TMT-A), reaction time (go no go test and DLRT), and attention (d2 test). The partial eta-square (η_p_^2^) values demonstrated small to large effect sizes in cognitive performance.

Additionally, for paired comparisons, the effect sizes in cognitive functioning for the exercise group were as follows: TMT variables – 0.20 for ‘TMT-A time’, 0.22 for ‘TMT-A errors’; 0.26 for ‘MOCA – memory’; 0.29 for ‘Eriksen flanker task’; d2 test- 0.32 for ‘Items processed’, 0.24 for ‘Items recognized correctly’, 0.19 for ‘Errors’, 0.32 for ‘Total efficacy’; DLRT - 0.26 for ‘SRT’, 0.29 for ‘SRT-DT’, 0.21 for ‘Mental calculations’ and 0.28 for the ‘CRT’.

No dropouts were recorded during the intervention period, and any missed sessions were rescheduled for compensation. Two compensation sessions were required. The Borg Scale, utilized to monitor participants’ perceived effort, averaged 6.7 points over the entire 8-week period. Furthermore, the program achieved a mean satisfaction rating of 4.3 points on the analog scale.

## Discussion

As the global population ages, it becomes increasingly important to develop approaches that promote health and overall well-being. Physical activity holds the potential to positively impact the functional status of older individuals. This study provides evidence that an 8-week psychomotor processing speed training program (conducted twice a week) is effective in enhancing various cognitive and physical abilities in older adults living in the community. Overall, notable changes were observed in the majority of variables within the experimental group. The study revealed a significant impact of participation in the exercise program, with effect sizes ranging from small to high. It is important to note that at baseline, participants in both groups had relatively low performances both physically and cognitively.

Notably, there were no dropouts during the intervention, and any missed sessions were compensated for, suggesting that the program was well-tolerated. Overall, participants expressed a high level of satisfaction (mean >4) with the sessions, as assessed using a 5-point Likert scale at the end of each session. Maintaining high participant satisfaction is crucial to keeping individuals active and engaged in such exercise programs. Additionally, adjustments in exercise intensity and specificity were made between sessions based on participants’ perceived effort, as measured by the Borg scale.

Processing speed has traditionally been trained using cognitive tasks, and there is a lack of data on comparable psychomotor approaches. Typically, conventional processing speed training is conducted on computers or with resources from other electronic devices (Bonnechère et al., 2021; Takeuchi & Kawashima, 2012). However, the approach to induce benefits in the rapid processing of information can be more diverse (Takeuchi & Kawashima, 2012). In this study, we aim to explore the use of movement-related exercises to intervene in components of processing speed, including perceptual speed, decision speed, psychomotor speed, and reaction time (Salthouse, 2000). In addition to induce cognitive benefits, the activities used were also planned for targeting physical fitness components.

It is important to highlight that traditional cognitive processing speed interventions primarily focus on improving cognitive abilities, such as processing speed, memory, and attention. In contrast, our innovative protocol adopts a completely different approach, seeking to enhance not only cognitive abilities but also a broad range of physical skills.

Regarding physical function, within-group comparisons between the baseline and post-intervention evaluations indicated improvements in strength, upper limb flexibility, aerobic capacity, agility, balance, and gait speed for the EG. Assuming that our intervention program involved multimodal exercises targeting different physical components, our data aligns with a literature review (C. Liu et al., 2017), which reported the effectiveness of multimodal exercise in increasing muscle strength, balance, gait speed, and chair stand. However, it is noteworthy that the previous review focused solely on physical aspects, whereas our study also emphasizes cognitive aspects. In a more extensive 24-week multimodal exercise program, incorporated dual-task exercises involving both cognitive and motor components, employing a psychomotor approach similar to ours. The authors also observed improvements in mobility (TUG) (Rosado et al., 2021). Another study utilizing a multimodal intervention program reported enhancements in the chair stand test, back scratch test, and timed up and go test (Shahtahmassebi et al., 2019), consistent with our findings. It is essential to highlight a key distinction between our study and conventional multimodal studies, as we specifically emphasize reaction time and the speed of task execution, emphasizing the completion of cognitive tasks in the shortest possible time.

Specifically, we observed the positive effects of psychomotor processing speed training on both upper and lower body strength, as assessed by the Senior Fitness Test (SFT) and Short Physical Performance Battery (SPPB). In all assessments, improvements exceeded 13% (Table 3), aligning with similar results obtained in other studies (Gianoudis et al., 2014). Additionally, crucial components for everyday life, such as agility (TUG), balance (SPPB), and gait speed (SPPB), also exhibited significant improvements due to our program. This outcome is consistent with findings from other studies, both of which demonstrated that integrating physical activity with cognitive training (dual-task) yields benefits in these capacities (Imaoka et al., 2019; Rosado et al., 2021). The effect sizes (η_p_^2^) for these capacities were estimated to be large.

As mentioned earlier, our intervention methodology was centered around dual-task performance, encompassing both cognitive and motor capacities. When examining studies involving simultaneous cognitive and physical stimulation, particularly dual-task activities, the results are generally consistent with those obtained in our study. Specifically, improvements are observed in various physical components such as strength, aerobic capacity, agility, and balance, alongside enhancements in cognitive functioning, including processing speed, attention, memory, and reaction time (Biazus-Sehn et al., 2020; Mai Ba & Kim, 2022; Nishiguchi et al., 2015; Phirom et al., 2020).

Among the comprehensive cognitive assessments, it is crucial to emphasize the advantages of our program in attention capacity, evaluated through the d2 test. We have identified notable benefits across all test parameters exceeding 10%. These findings align with the outcomes reported in a 10-week multimodal psychomotor intervention conducted in nursing homes (Pereira et al., 2018). According to the study, participants in the experimental group displayed improved selective and sustained attention, along with enhanced visual scanning speed (Pereira et al., 2018). Moreover, in a sample resembling ours, consisting of community-dwelling older adults with a mean age of 75.4 years, propose that a psychomotor multimodal intervention, centered on dual-task activities, effectively stimulates attention capacity (Rosado et al., 2022). As previously mentioned, the TMT-A assesses visual scanning, graphomotor speed, and executive functioning. Our data revealed that the EG improved performance in TMT-A post-intervention, requiring an average of −9% less time to complete the test. Similarly, a randomized controlled trial showed that multicomponent exercise (cognitive and aerobic training) could lead to significant enhancements in TMT-A scores (Imaoka et al., 2019). Another study demonstrated that an 8-week regimen of combined cognitive and physical exercise, comprising three sessions per week, led to a significant improvement in performance on the TMT-A and enhanced executive functions in older adults. This suggests that the integration of exercise and cognitive training may yield broader benefits when compared to isolated computerized cognitive training (ten Brinke et al., 2020).

Our data also reveal a positive impact of the intervention on reaction time, including in SRT-DT. Following the 8-week program, participants exhibited increased speed and reduced response time to stimuli. Comparable evidence was identified in a study involving a multimodal psychomotor program, where participants demonstrated improvements in reaction time and dual-task performance (Rosado et al., 2021).

The SFT-chair sit and reach test, the Arm Ruler Positioning test, the MOCA-memory, and the Eriksen flanker task did not yield statistically significant changes within the groups. However, regarding the chair sit and reach test and the Arm Ruler Positioning test, ANCOVA analysis revealed statistically significant differences between the groups, with estimated medium effect sizes (>0.06). Nonetheless, concerning the Eriksen flanker task, despite the absence of changes within the group and no significant differences in the ANCOVA analysis, it can be observed that when we examine the effect size (Table 4), it was estimated to be 0.09 (medium). Although intra-group changes were apparently insignificant, the data suggest that participants in the experimental group (EG) were able to enhance their ability to identify specific characteristics while abstracting from irrelevant components in the task. Furthermore, our findings are supported by previous studies (ten Brinke et al., 2020; Themanson & Hillman, 2006) which have demonstrated that the combination of exercise and cognitive stimulation can significantly improve performance on the flanker task.

It’s worth noting that concerning the collected data from the SFT regarding physical fitness, the initial general capacities of the participants in the exercise group were notably low, typically demonstrating performances between the 10th and 25th percentile (Marques et al., 2014). This factor may have ultimately contributed to the positive outcomes observed in the physical capabilities of participants in the intervention program.

The present study has significant strengths. First, it provides data on a relatively understudied intervention model: an innovative psychomotor processing speed program tailored for community-dwelling older adults. Furthermore, our evidence highlights that a relatively brief intervention period can yield a numerous of physical and cognitive benefits. Additionally, even with shorter weekly exercise periods than suggested by WHO guidelines, the results were quite promising for this population, suggesting the possible relevance of this type of intervention program (World Health Organization, 2020). However, the study also has some limitations. Firstly, it is a non-randomized controlled trial conducted with a convenience sample. Secondly, no follow-up was performed. Thirdly, the benefits observed between aerobic and anaerobic exercises were not distinguished; future studies should examine this further. Fourthly, we did not compare our intervention with a processing speed program focused solely on cognitive stimulation, which would allow for an analysis of the distinct potentials and advantages of each stimulation methodology. Future work could address this. Also, future studies should explore the effects of psychomotor processing speed interventions on community-dwelling older adults with larger samples and extended intervention periods. This approach would provide a more comprehensive evaluation of the intervention’s impact.

## Conclusion

The current study demonstrated the effectiveness of an 8-week movement-based processing speed training program in enhancing various physical and cognitive abilities. The integration of psychomotor processing speed training, merging both physical and cognitive stimulation, has the potential to positively impact the individual’s general level of functioning. This approach should be actively promoted among community-dwelling older adults.
